# Shape and Composition Control of Monodisperse Hybrid Pt-CoO Nanocrystals by Controlling the Reaction Kinetics with Additives

**DOI:** 10.1038/s41598-017-04211-9

**Published:** 2017-06-20

**Authors:** Hyunje Woo, Eunji Kim, Jun-Hyuk Kim, Su-Won Yun, Ji Chan Park, Yong-Tae Kim, Kang Hyun Park

**Affiliations:** 1 Department of Chemistry and Chemistry Institute for Functional Materials, Pusan National University, Busan, 46241 Korea; 2 Hybrid Materials Solution National Core Research Center (NCRC), Pusan National University, Busan, 46241 Republic of Korea; 3 Department of Energy System, School of Mechanical Engineering, Pusan National University, Busan, 46241 Korea; 4 Clean Fuel Laboratory, Korea Institute of Energy Research, Daejeon, 34101 Korea

## Abstract

Here, we report the effect of Fe(CO)_5_ additives in the synthesis of branched Pt-CoO nanowires (NWs) and core@shell concave nanocubes (NCs), in a one-pot system. Key to the success of this synthesis is control over the shape of the Pt seeds by controlling the quantity of Fe(CO)_5_ additive. In the absence of Fe(CO)_5_, branched Pt-CoO NWs were synthesized through the attachment of small Pt seed particles, followed by the growth of CoO by deposition. On the other hand, Pt@CoO concave NCs were obtained in the presence of Fe(CO)_5_ because of the stronger adsorption of Co on the Pt (100) surfaces than on the closely packed (111) surfaces. Also, various other conditions including the control of reducing agents, precursor concentrations, and stabilizing agents, were used to verify the effects of reaction kinetics on the synthesis of Pt-CoO nanoparticles. Compared to Pt/graphene oxide (GO) catalyst, branched Pt-CoO NWs supported on GO showed enhanced specific activity toward the oxygen reduction reaction (ORR).

## Introduction

Recently, several attempts have been made to develop hybrid Pt-CoO nanoparticles (NPs) due to an increasing demand for Pt-based catalysts with enhanced electrocatalytic activities and for magnetic applications^1, 2^. Aliviasatos *et al*. developed Pt@CoO yolk-shell nanostructures through the decomposition and reduction of metal precursors through the Kirkendall effect^3^. Tao and co-workers reported Pt_m_Co_m’_/CoO_1−x_ nanorods as efficient catalysts for the water-gas shift reaction^4^.

Generally, the morphology control of noble metal NPs is governed by two pathways^5, 6^: thermodynamic or kinetic control in the solution state. The thermodynamic control is driven by the chemical potential, which is dependent on the temperature and supersaturation of the solution. In contrast, under the kinetic control pathway, various morphologies of different dimensions can be obtained by altering the reaction conditions. Among them, additives such as halides, metal ions, and metal salts have proved to be efficient directors especially for metal NP morphology. This demonstrates that such additives can control the growth rate of specific facets leading to desirable metal NP morphologies^5, 7–9^. Sun *et al*. reported well-defined Pt nanocubes (NCs) with a trace amount of Fe(CO)_5_^10^; the trace amount of Fe(CO)_5_ was key to achieving shape control. However, it remains greatly challenging to control the morphology of hybrid Pt-CoO NPs using small amounts of additives. Our ongoing interest in controlling the morphology of metal-metal oxide NPs motivated us to investigate the synthesis of Pt-CoO NPs using Fe(CO)_5_ additives via a facile approach.

In the field of catalysis, hybrid Pt-CoO nanostructures, as electrochemical catalysts, have attracted much attention due to the formation of interfacial regions between the platinum and CoO^2^. In addition to forming hybrid nanostructures, it is also crucial to use a unique support such as graphene oxide (GO) to enhance catalytic activity and stability, as there often exists a charge transfer across the metal/GO interface^11^. GO-supported Pt-CoO NP nanocomposites as catalysts for the oxygen reduction reaction (ORR) have been not reported in the literature.

**Figure 1:**
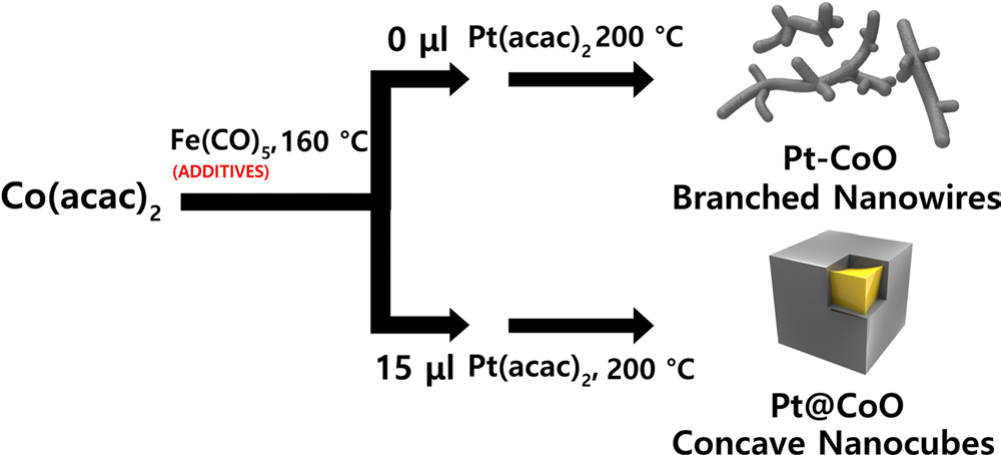
Synthetic scheme of Pt-CoO nanostructures.

In this work, we report the facile synthesis of monodisperse hybrid Pt-CoO NPs, and study their catalysis of the ORR under fuel cell conditions. The selective formation of Pt-CoO nanostructures was attained by introducing different amount of Fe(CO)_5_ additive (Fig. 1). To our knowledge, this is the first report on the role of reaction kinetics in the selective formation of Pt-CoO NPs. It not only provides a robust route to Pt@CoO concave NCs but also offers an insight into kinetic control in the shape-controlled synthesis of Pt-CoO nanocrystals. Electrochemical studies showed that the GO-supported Pt-CoO branched NWs catalyzed the ORR in a 0.1 M HClO_4_ aqueous solution, and had a specific activity higher than that of the Pt/GO catalyst.

## Results and Discussion

**Figure 2:**
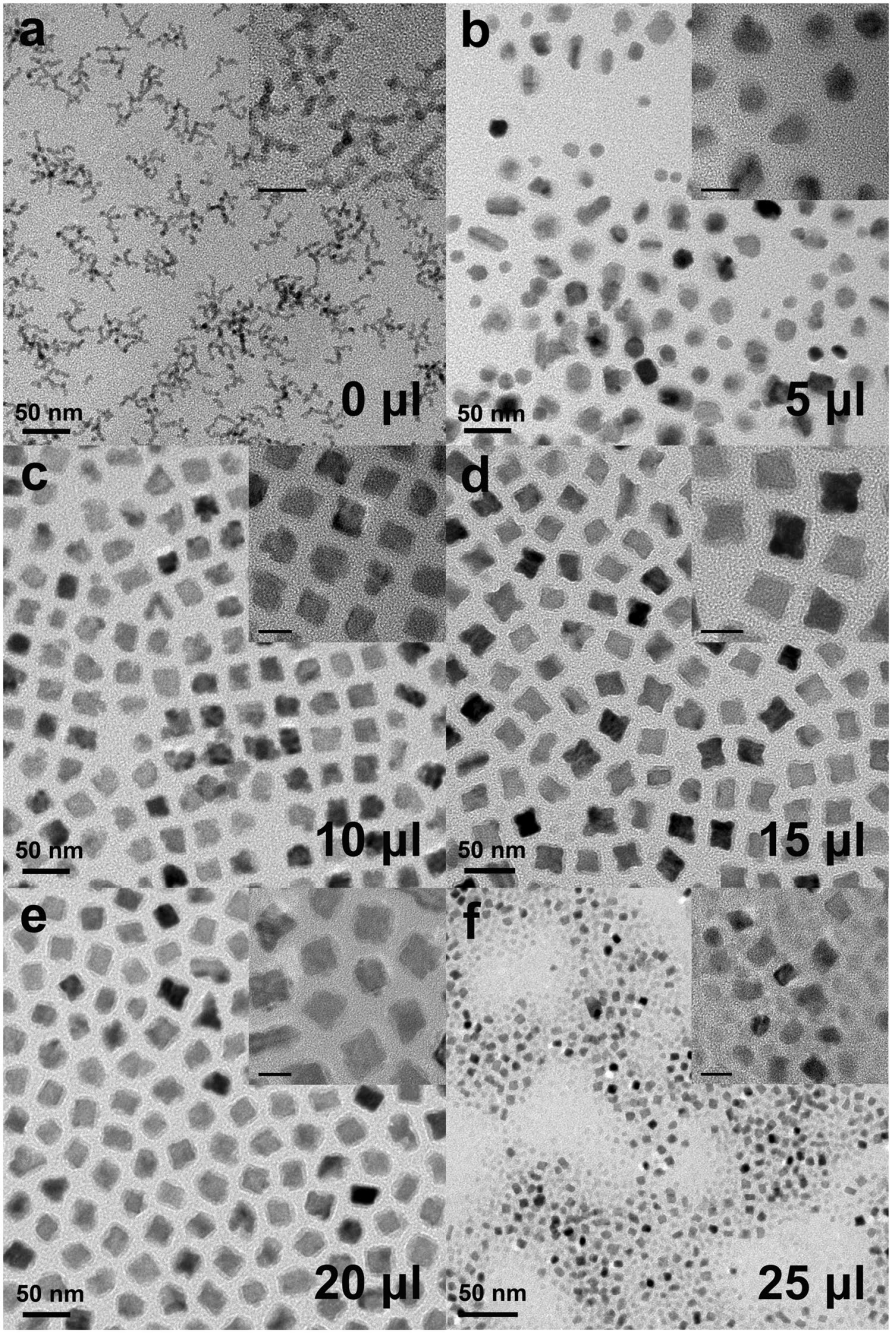
TEM images of Pt-CoO NPs obtained by changing the amount of Fe(CO)_5_ (0~25 μl) (**a**~**f**). The bars in the inset represent 10 nm.

**Figure 3:**
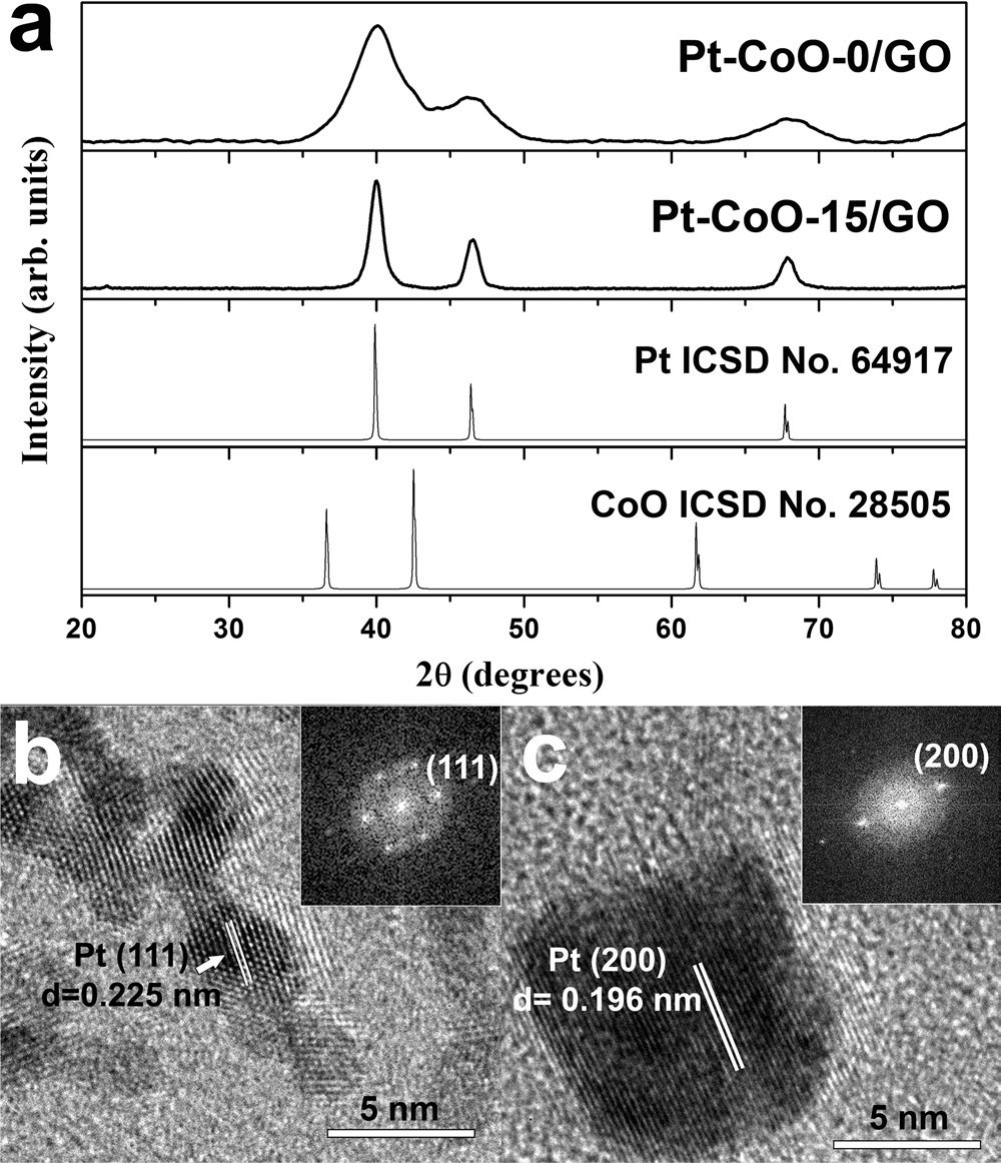
XRD patterns (**a**) and HR-TEM images of Pt-CoO-0/GO (**b**) and Pt-CoO-15/GO (**c**).

**Figure 4:**
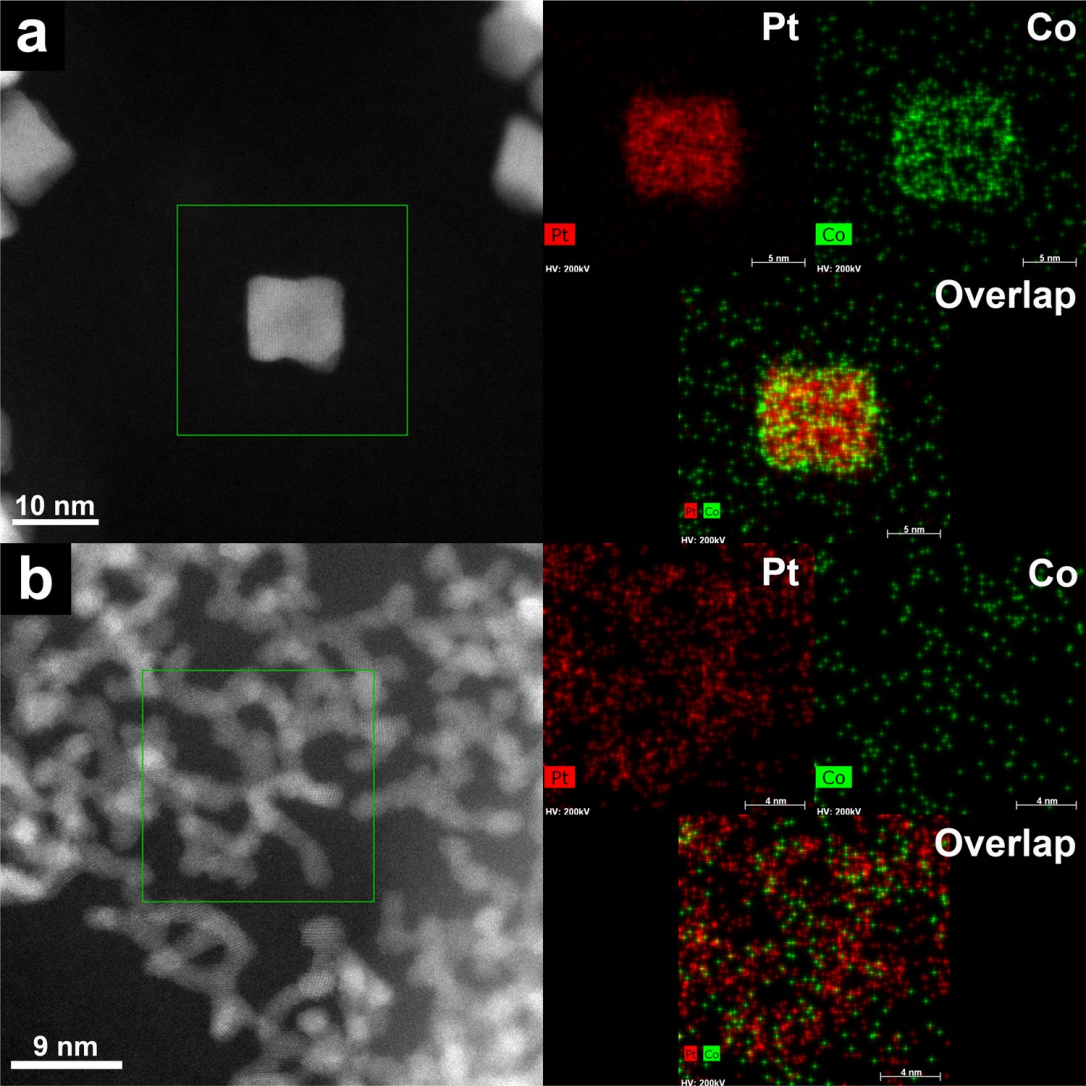
HAADF-STEM image and elemental mapping of Pt-CoO-15/GO **(a**) and Pt-CoO-0/GO (**b**).

Figure 2 shows transmission electron microscopy (TEM) images of Pt-CoO-*n* nanostructures obtained by changing the amount of Fe(CO)_5_; *n* denotes the added amount of Fe(CO)_5_ in the reaction mixture (0 to 25 μl). In the absence of Fe(CO)_5_, Pt-CoO-0 branched nanowires (NWs) were obtained through the oriented attachment of small Pt NPs following CoO deposition on Pt (Fig. 2a). Interestingly, when certain amounts of Fe(CO)_5_ were introduced, changes in the Pt-CoO NP morphology were confirmed, and these changes depended on the amount of Fe(CO)_5_ added. By increasing the Fe(CO)_5_ amount from 5 μl to 15 μl, while keeping other conditions identical, we found that the products changed from irregular-shaped ones to core@shell concave cubes structures (Fig. 2b–d). When the Fe(CO)_5_ amount was increased to 20 μl, the morphology (Fig. 2e) was almost unchanged from that of the concave nanocubes (Fig. 2d). However, the uniformity of NPs was drastically decreased when 25 μl Fe(CO)_5_ was used (Fig. 2f). These results indicated that the composition of the final products could be readily manipulated, shifting from branched NWs to core@shell structures, by the introduction of different amounts of Fe(CO)_5_. Low-magnification TEM images of branched Pt-CoO-0 NWs and Pt-CoO-15 and Pt-CoO-20 NPs are shown in Fig. S1, demonstrating nearly 100% purity [with an average diameter of 12 nm (Pt-CoO-15, Fig. S1b) and 13 nm (Pt-CoO-20, Fig. S1c)]. As a control experiment, Pt NPs were synthesized without the addition of Co(acac)_2_ (Fig. S2), showing the nanocube morphology. The crystal structure of the samples was characterized by X-ray powder diffraction (XRD), as shown in Fig. 3a. All diffraction peaks in the XRD pattern are characteristic of the face-centered cubic (fcc) structure of Pt (ICSD #64917). The high-resolution TEM (HR-TEM) images shown in Fig. 3b,c also demonstrate the formation of highly crystalline Pt particles, with Pt-CoO-15 nanocubes and Pt-CoO-0 NWs lattice fringes. The line spacing of 0.225 nm could be indexed to Pt (111) reflections of the fcc structure. The presence of crystalline CoO was not confirmed, indicating that the cobalt oxides form an amorphous phase. This is explained by the high solution temperatures (>200 °C) and hydrothermal conditions generally required for crystalline CoO syntheses^12^. The high-angle annular dark-field scanning TEM (HAADF-STEM) image and elemental mappings of Pt and Co show the core@shell Pt-CoO-15 nanocubes (Fig. 4a) and branched Pt-CoO-0 NWs (Fig. 4b) structures. The Pt-CoO-15 nanocubes contain CoO (green) in the shell and Pt (red) in the core. Pt and CoO are dispersed in all areas of branched Pt-CoO-0 NWs, confirming the hybrid Pt-CoO structure.

**Figure 5:**
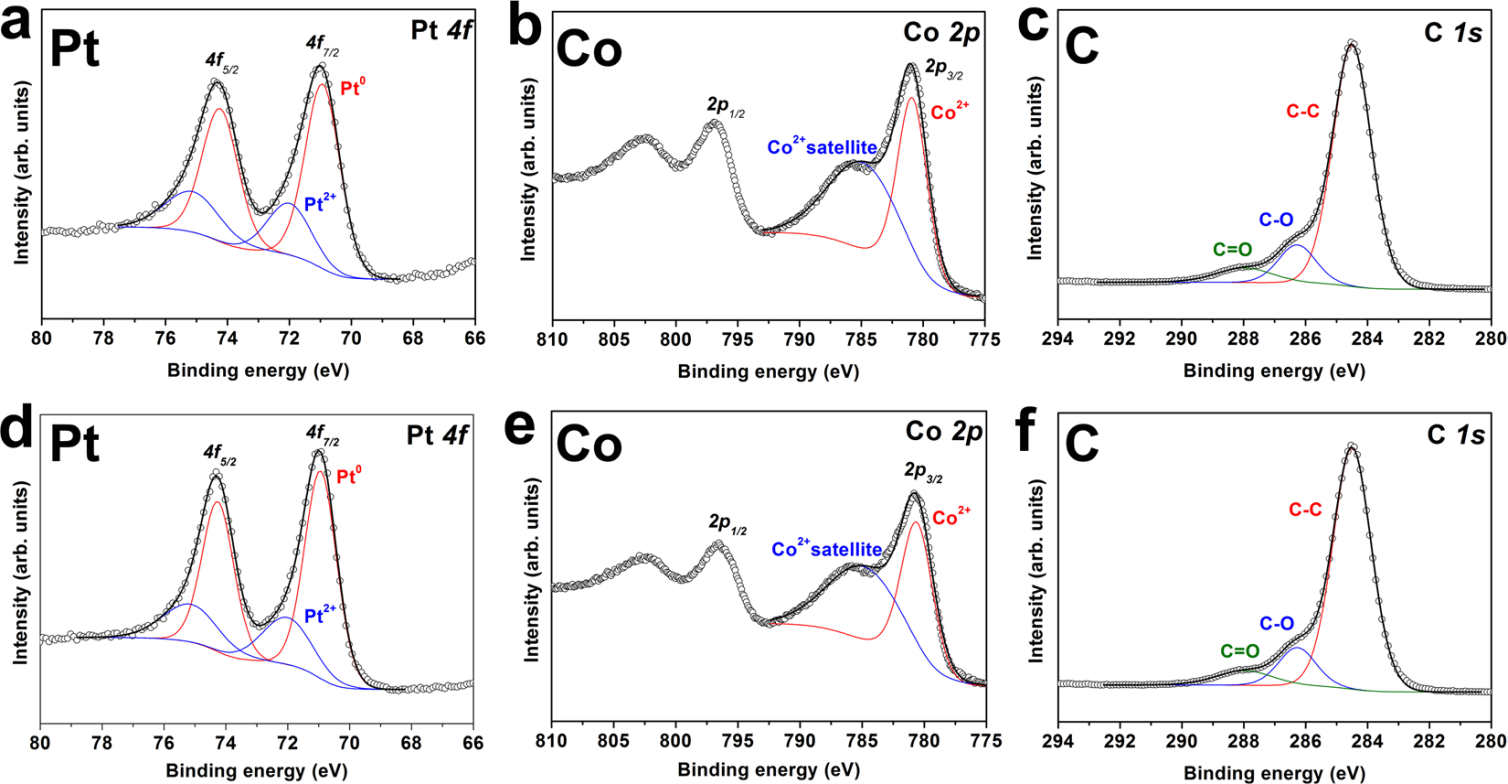
XPS spectra of Pt-CoO-15/GO (**a**,**b**,**c**) and Pt-CoO-0/GO (**d**,**e**,**f**).

Elemental analysis results of Pt-CoO-15/GO and Pt-CoO-0/GO were confirmed by using X-ray photoelectron spectroscopy (XPS) (Fig. 5). For both samples, the binding energies of the Pt 4f_7/2_ and Pt 4f_5/2_ doublet, shown in Fig. 5a,d, are characteristic of metallic Pt^13, 14^. The Co 2p_3/2_ peak can be deconvoluted into two components (Fig. 5b,e). In the case of Pt-CoO-15, the most intense peak at 780.6 eV is assigned to Co 2p_3/2_, and the one at 784.8 eV corresponds to a Co^2+^ satellite. Both peaks indicate the presence of CoO on the surface of Pt nanostructures. The binding energy of the C-C is assigned at 284.5 eV and shifts of +1.6 and +3.5 eV are typically assigned for the C–O and C = O functional groups, respectively (Fig. 5c,f)^15^.

**Figure 6:**
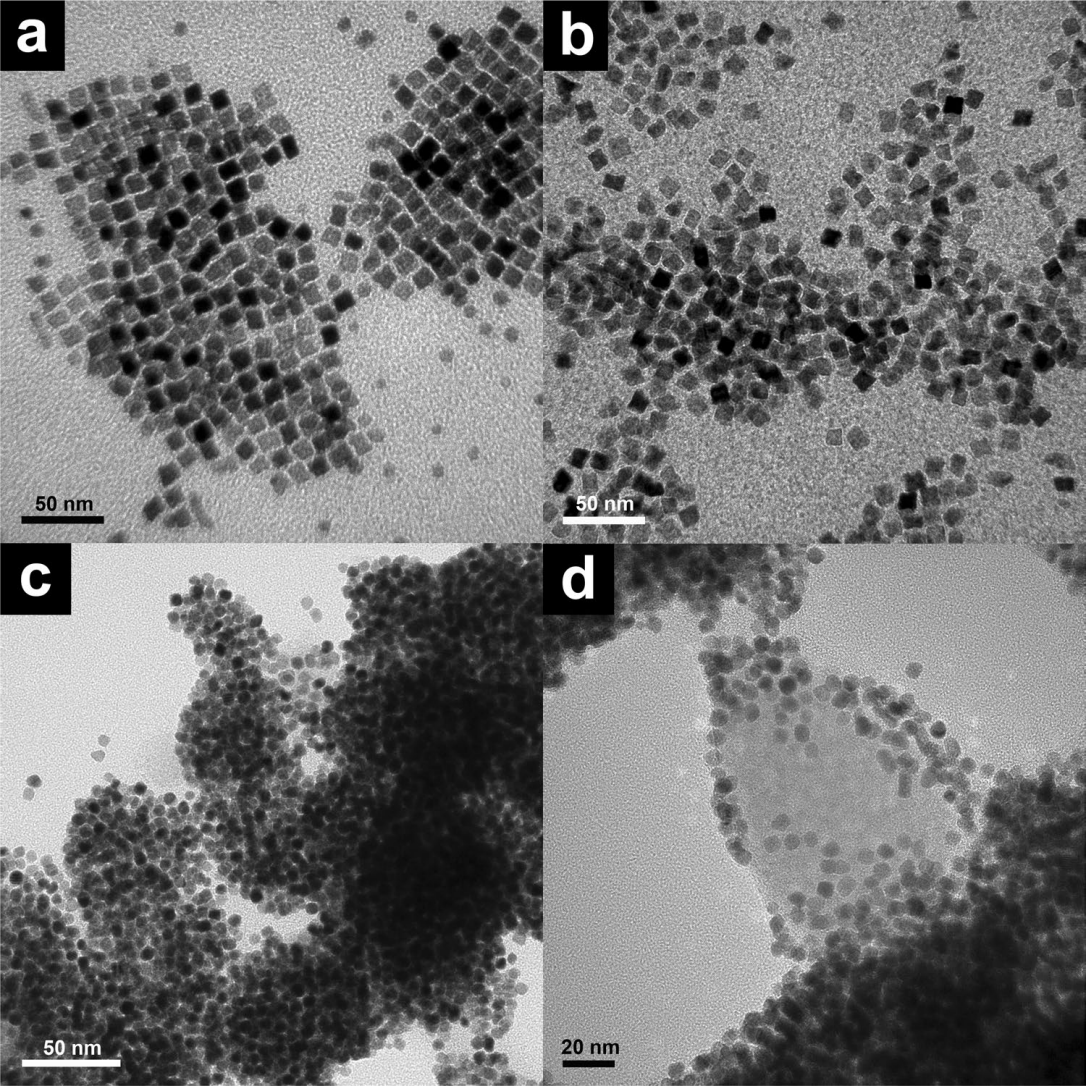
TEM images of Pt-CoO NPs obtained by adding Cr(CO)_6_ (**a**) and W(CO)_6_ (**b**) instead of Fe(CO)_5_; using excess CO gas (**c**) instead of Fe(CO)_5_.

To confirm the effects of foreign metals in the synthetic process, Cr(CO)_6_ and W(CO)_6_ were added instead of Fe(CO)_5_ with the same mole ratio; this resulted in nanocube morphologies without a shell structure (Fig. 6a,b). Cr(CO)_6_ and W(CO)_6_ additives did not direct the formation of a core@shell Pt-CoO structure, and this was only possible using Fe(CO)_5_. Carbon monoxide (CO) reducing/binding effects were also studied. It is known that Pt nanocube formation can be facilitated by the presence of CO. At a fixed ratio of oleic amine (OAm)/oleic acid (OA), the adsorption of CO on Pt atoms is stronger on the (100) surfaces than the closely packed (111) surfaces^16, 17^. In this work, the presence of CO molecules from Fe(CO)_5_ was the key to achieving a cube structure. However, when an excess amount of CO gas was injected to the reaction mixture instead of Fe(CO)_5_, only small particles were synthesized, demonstrating that CO molecules function both as a reducing agent and a capping ligand (Fig. 6c,d)^16^. It can be concluded that a suitable amount of both Fe ions and CO molecules is provided from Fe(CO)_5_, and that this is important in synthesizing core@shell Pt@CoO nanostructure.

A detailed mechanistic understanding of the structural evolution of the core@shell Pt@CoO structure was obtained by examining the temporal images of reaction intermediates. Initially, spherical CoO seed nanoparticles were obtained at 200 °C, before the injection of the Pt(acac)_2_ solution (Fig. S3a). Interestingly, the core Pt nanocube started to form after the injection of Pt(acac)_2_ but prior to the deposition of CoO particles (Fig. S3b). It seems that the synthesis involves the formation of Pt nanocubes and the subsequent deposition of CoO on the Pt nanocubes, which results in Pd@CoO core@shell structures.

**Figure 7:**
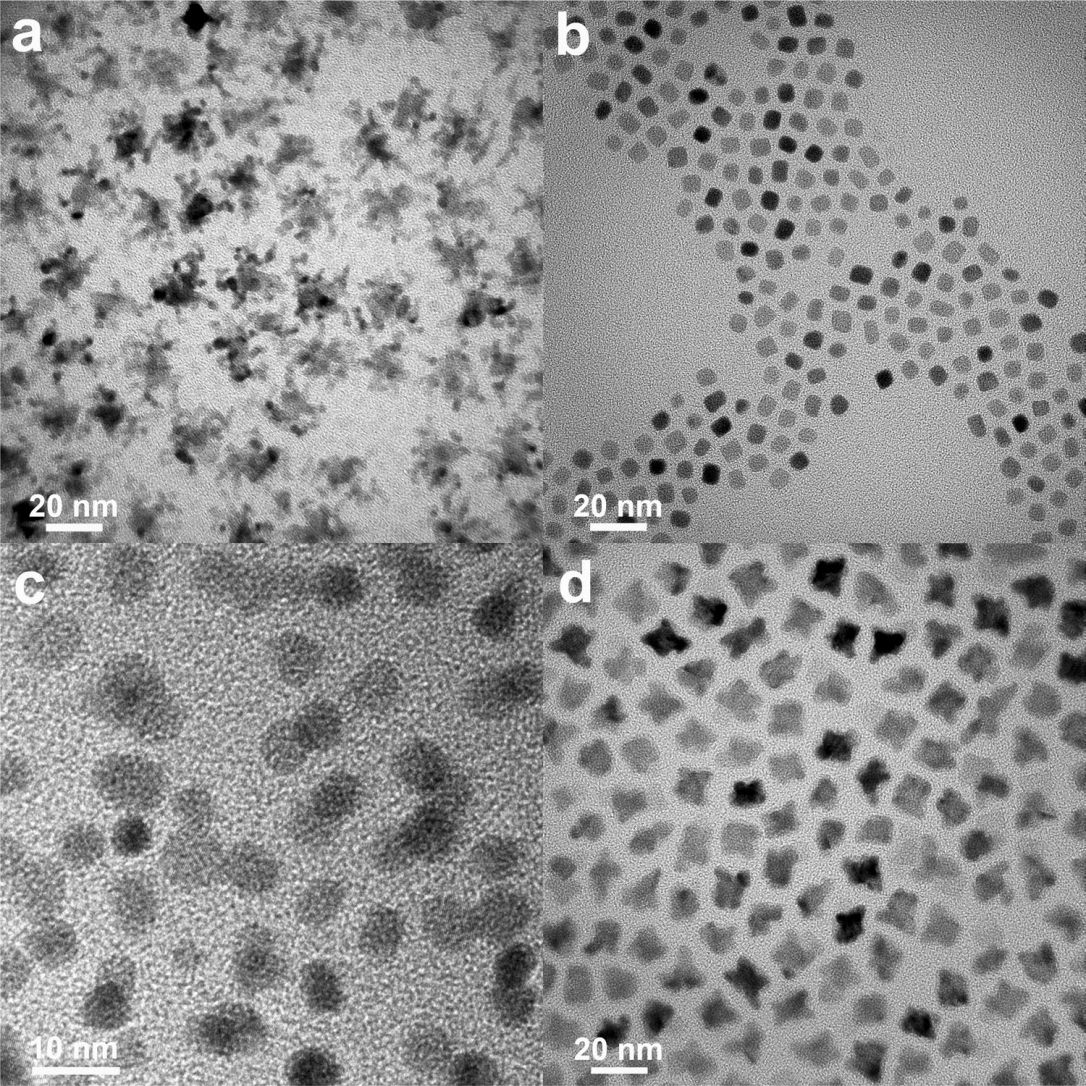
TEM images of Pt-CoO NPs obtained by using different quantity of OAm 2 ml (**a**) and 0.5 ml (**b**); Co(acac)_2_ 1 mmol (**c**) and 0.25 mmol (**d**).

To verify the effects of reaction kinetics on the synthesis of Pt-CoO NPs, a set of control experiments were conducted. Since the reducing power can be controlled through the reducing agent (OAm), different amounts of OAm were introduced into the solution to adjust the reduction kinetics. Interestingly, when the initial amount of OAm was increased to 2 ml, dendritic nanostructures consisting of different numbers of small particles were produced, revealing a process of self-assembly of small particles (Fig. 7a). If the reduction rate was extremely fast, the formation of a huge number of nuclei could occur, contributing to the self-assembly process^8^. On the other hand, the smaller Pt nanocubes were only obtained when the initial amount of OAm was 0.5 ml, owing to slower reduction kinetics (Fig. 7b). Control of precursor concentrations is also crucial for directional growth under a kinetically controlled regime, due to monomer concentrations and chemical potentials. For example, Yang *et al*. reported branched Pt NPs formed by employing a high precursor concentration^18^. In our experiment, the morphology of the synthesized products could be dramatically changed by varying the amount of Co(acac)_2_ used in the synthesis. When the quantity of Co(acac)_2_ was increased to 1 mmol, the high chemical potential of CoO particles resulted in the attachment of small NPs (Fig. 7c). Interestingly, more concaved Pt@CoO nanocubes were produced.

With low amounts of the Co(acac)_2_ precursor (0.25 mmol) (Fig. 7d). Additionally, the use of a combination of stabilizing agents with different functional groups, such as acids and amines, which have different binding strengths, provided control over NP morphology. Since OA is a weaker surfactant for Pt than OAm, it facilitates the growth of the particles into cubes^10^. By replacing OA with an equivalent molar amount of adamantaneacetic acid, while keeping all other reaction conditions the same, we could produce irregular Pt-CoO nanocrystals as it was not enough to bind surface atoms due to their steric hindrance effect (Fig. S4a). In the absence of OA, the Pt@CoO NPs were significantly more round, indicating that OA played a key role in sharpening the edges of the polyhedra during the synthesis (Fig. S4b)^19^. Figure S5 shows the nanocube and irregularly shaped Pt@CoO nanocrystals obtained at a reaction temperature of 160 °C, demonstrating that it is not enough to reduce Pt(acac)_2_ precursors at a low temperature.

**Figure 8:**
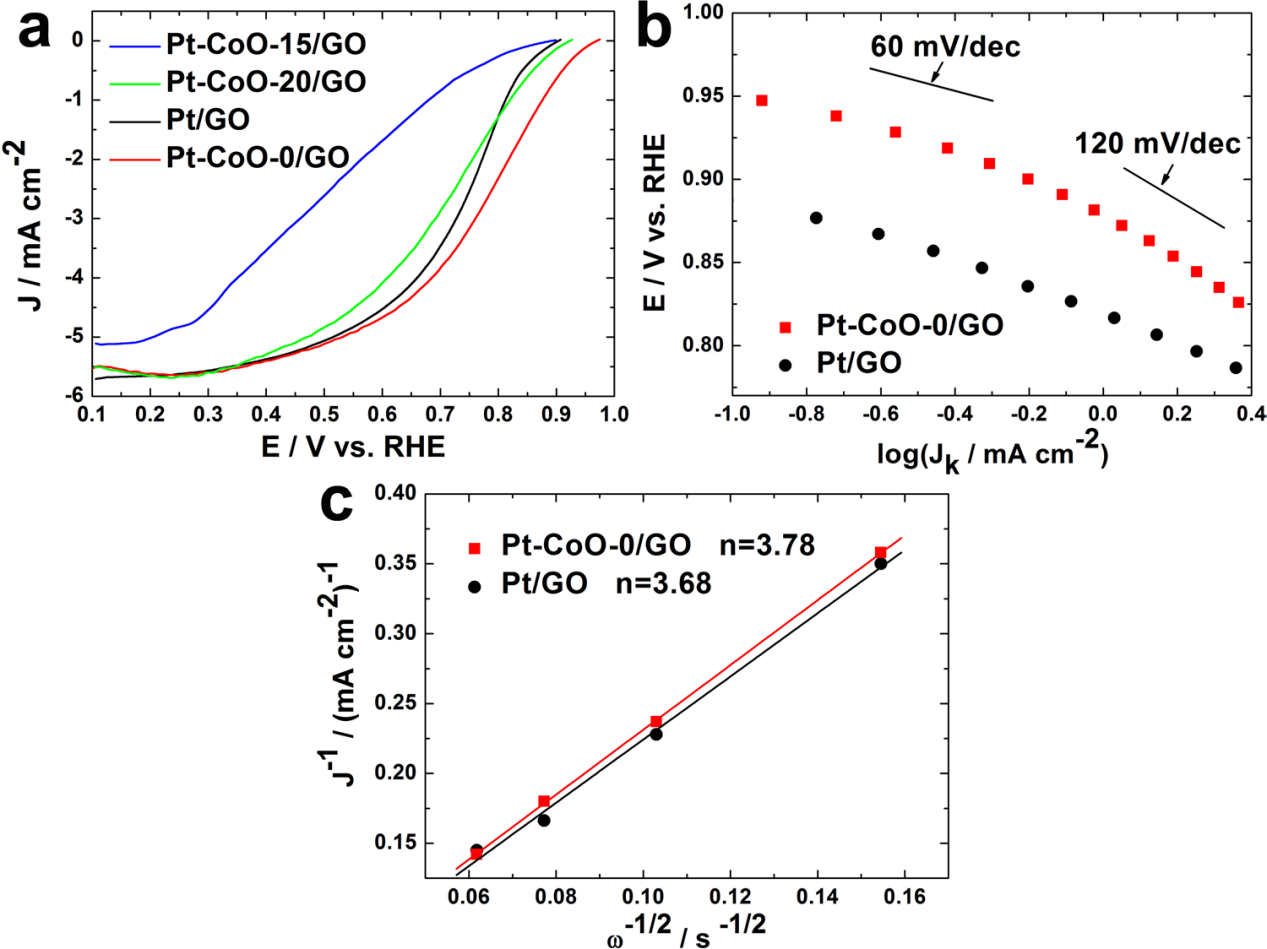
Comparison of electrochemical activities of Pt/GO, Pt-CoO-0/GO, Pt-CoO-15/GO, Pt-CoO-20/GO. (**a**) ORR polarization curves at 1600 rpm. (**b**) Tafel plots for the ORR measured at 1600 rpm. (**c**) Koutecky-Levich plots for the ORR at 0.3 V vs. RHE measured at 400, 900, 1600, and 2500 rpm.

The ORR activity was investigated using a thin-film rotating disk electrode (TF-RDE). Figures S6 and 8 show the cyclic voltammograms and ORR polarization curves for Pt/GO, Pt-CoO-0/GO, Pt-CoO-15/GO, and Pt-CoO-20/GO. Before conducting the performance of electrocatalysis, all of catalysts was already under heat treatment (3% H_2_/Ar) during 3 h at 300 °C to eliminate adsorbed OAm on Pt. The FT-IR data shows decrease the intensity of methyl stretching (2920~2850 cm^−1^) of OAm, demonstrating the remove of OAm on Pt surface after heat treatment (Fig. S7). The ORR curves were measured at a scan rate of 10 mV∙s^−1^ at 1600 rpm (Fig. 8a). The highest onset potential was found with Pt-CoO-0/GO (0.98 V), followed by Pt-CoO-20/GO (0.93 V), and the activities for Pt/GO and Pt/CoO-15/GO were identical (0.90 V). The onset potential of Pt-CoO (0.97 V) synthesized by addition of extra CO gas was also higher than that of Pt/GO because of monodispersed small Pt nanoparticles (Fig. S8). In the kinetics- and diffusion-controlled region, significant ohmic loss was observed for all the Pt-CoO/GO samples due to the core@shell structures; the surface-exposed CoO considerably blocks the active surface of the Pt in Pt-CoO-15/GO, which shows very low ORR activity. In Pt-CoO-0/GO, the excellent ORR performance is attributed to the high surface exposure of Pt atoms as shown by TEM. The Tafel slopes for the ORR kinetic current of Pt/GO and Pt-CoO-0/GO are shown in Fig. 8b. In general, Pt metal assumes two different speciation: pure Pt at a lower potential and Pt-CoO mixture at a higher potential, resulting in the two polarization curves (lower Tafel slope: 60 mV dec^−1^ and 120 mV dec^−1^)^20^. The measured Tafel slope was lower than these values due to the surface blockage by CoO. A Koutecky-Levich plot for the ORR in Pt/GO and Pt-CoO-0/GO is shown in Fig. 8c. The plot exhibits the inverse current density (J^−1^) as a function of the inverse square root of the rotating rate (ω^−1/2^) and demonstrates first-order kinetics with respect to molecular oxygen^21^. The slope was calculated by linear fitting, and a 4-electron transfer reaction was confirmed for the n value of about 4. The n value was calculated as 3.78 for Pt-CoO-0/GO and 3.68 for Pt/GO, indicating almost 4-electron transfer process for the ORR. The methanol poisoning effect in ORR activity was investigated by amperometry measurements at Pt-CoO-0/GO electrodes at a fixed potential of 0.05 V (with a rotation rate of 1600 rpm) in 0.1 M HClO_4_ for 2000 s (Fig. S9). The first 1000 s of the amperometry was performed under oxygen saturated conditions and next 1000–2000 s was performed with 3 M methanol addition under oxygen saturated conditions. Pt-CoO-0/GO electrodes displayed slightly decrease of current density even after addition of 3 M methanol, demonstrating superior methanol tolerance^22^. Long-term stability test was also conducted to investigate the morphology of Pt-CoO-0/GO after potential cycling between 0.6 V and 1.1 V^23^. Pt-CoO-0/GO catalyst maintained their well-defined branched nanowires after long-term stability test, exhibiting good stability and recyclability against the acidic reaction conditions (Fig. S10).

## Conclusion

A method for the one-pot synthesis of hybrid Pt-CoO nanostructures through controlled reduction of metal precursors was successfully developed. Fe(CO)_5_ additives were confirmed to be crucial for shape control. The associated morphology evolution was shown to involve the initial formation of Pt seeds followed by subsequent growth of CoO. The effects of reaction kinetics on the synthesis of Pt-CoO NPs were found by varying the synthetic conditions that included control of reducing agents, precursor concentrations, and stabilizing agents. Among synthesized catalysts, Pt-CoO-0/GO catalyst showed the highest specific activity toward the ORR, and the same concept may be applicable to other multimetallic NP systems for electrocatalysis.

## Method

### Characterization

The morphology of each sample was characterized by TEM (FEI TALOS F200X operated at 200 kV, Pusan National University) by placing a few drops of the corresponding colloidal solution on carbon-coated copper grids (200 mesh, F/C coated, Ted Pella Inc., Redding, CA, USA). The XRD patterns were recorded on a Rigaku D/MAX-RB (12 kW) diffractometer. XPS (Theta Probe, Thermo) was employed to measure the structural and chemical properties of the nanocomposites. A three electrode electrochemical cell with a potentiostat (Biologic VSP) was used to evaluate Pt-CoO/GO and Pt/GO samples.

### Synthesis of hybrid Pt-CoO NPs

0.129 g of Co(acac)_2_ was mixed with 10 mL 1-octadecene (ODE), 1 mL OA and 1 mL OAm. After being placed under vacuum at 100 °C for 1 h, the formed solution was slowly heated from 60 °C to 120 °C in 10 min. After 30 min of heating at 120 °C, under a blanket of argon, small amount (0.1 ml) of Fe(CO)_5_ solution [prepared by mixing (0.05~0.25 ml) Fe(CO)_5_ with 1 mL ODE] was added to this solution. The temperature was raised to 200 °C (4 °C/min) and kept at this temperature for 30 min. Then, Pt(acac)_2_ solution [Pt(acac)_2_ with ODE (4 ml) and OAm (1 ml) solution] was added and the mixture was kept at this temperature for 3 h. The solution was cooled down to room temperature. The product was separated by centrifugation with ethanol and hexane. The product was then dispersed in hexane.

### Synthesis of Pt-CoO/GO

Pt-CoO solution dispersed in 20 mL of hexane was added into 20 mL of DMF solution of G (2.5 mg/mL) and the mixture was sonicated for 1 h. After washing, the powder was then suspended in 40 mL of acetic acid and the suspension was heated at 70 °C for overnight to remove the surfactants around nanoparticles. The product was then centrifugated (8000 rpm 10 min), and was washed with ethanol two times before they were dried.

### Electrochemical measurements

All electrochemical tests were conducted at room temperature and ambient pressure. Well-dispersed samples were deposited on a 5 mm in diameter of glassy carbon (GC) electrode that was used as the working electrode. A platinum wire and a Ag/AgCl electrode are used as the counter and reference electrodes, respectively. The 0.1 M HClO_4_ at pH 1.0 was used as an electrolyte. All of catalysts was under heat treatment (3% H_2_/Ar) during 3 h at 300 °C to eliminate adsorbed OAm on Pt. The samples were mixed with 89.6 ml of distilled water, 10 ml of isopropanol, and 0.4 ml of Nafion solution. The ink of 30 μl dropped onto the working electrode (40 μg_metal_/cm^2^) surface to enhance the bond on the electrode surface. The durability tests were carried out at room temperature in O_2_-saturated 0.1 M HClO_4_ solutions, with a cyclic potential sweep between 0.6 and 1.1 V (vs RHE) at a sweep rate of 50 mV/s for 4000 cycles.

## Electronic supplementary material

Shape and Composition Control of Monodisperse Hybrid Pt-CoO Nanocrystals by Controlling the Reaction Kinetics with Additives
