# Supplementary material for: Shape and Composition Control of Monodisperse Hybrid Pt-CoO Nanocrystals by Controlling the Reaction Kinetics with Additives
Source: Sci Rep. 2017 Jun 20;7:3851. doi: 10.1038/s41598-017-04211-9 (PMC5478655; doi:10.1038/s41598-017-04211-9)
Supplement: Supplementary file 1 — Shape and Composition Control of Monodisperse Hybrid Pt-CoO Nanocrystals by Controlling the Reaction Kinetics with Additives [file 41598_2017_4211_MOESM1_ESM.pdf]

Supporting Information

Shape and Composition Control of Monodisperse Hybrid Pt-CoO Nanocrystals by Controlling the Reaction Kinetics with Additives

Hyunje Woo[1,2], Eunji Kim[1], Jun-Hyuk Kim[2], Su-Won Yun[3], Ji Chan Park[4], Yong-Tae Kim[3] & Kang Hyun Park[1,*]

[1] Department of Chemistry and Chemistry Institute for Functional Materials, Pusan National University, Busan 46241, Korea.
Phone: (+82)-51-510-2238; Fax: (+82)-51-980-5200; e-mail: chemistry@pusan.ac.kr
[2] Hybrid Materials Solution National Core Research Center (NCRC), Pusan National University, Busan 46241, Republic of Korea.
[3] Department of Energy System, School of Mechanical Engineering, Pusan National University, Busan 46241, Korea.
[4] Clean Fuel Laboratory, Korea Institute of Energy Research, Daejeon, 34101, Korea.

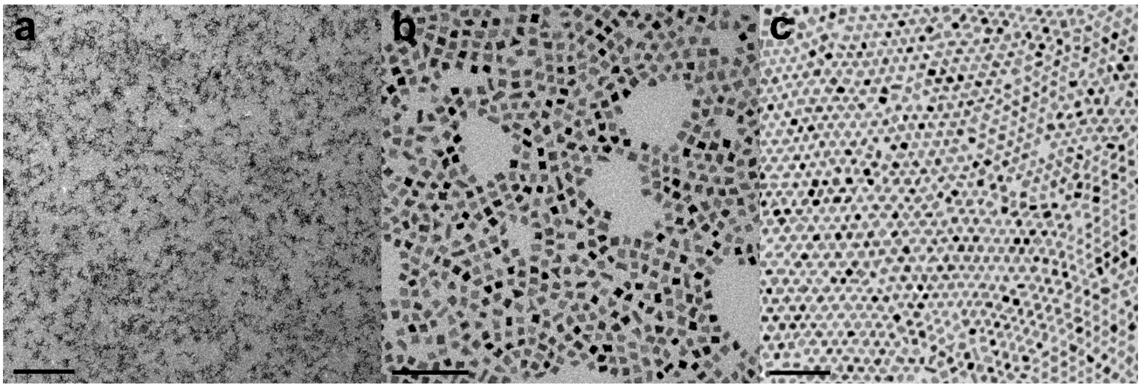

Figure S1. Low resolution TEM images of Pt-CoO-0 (a), Pt-CoO-15 (b) and Pt-CoO-20 (c).

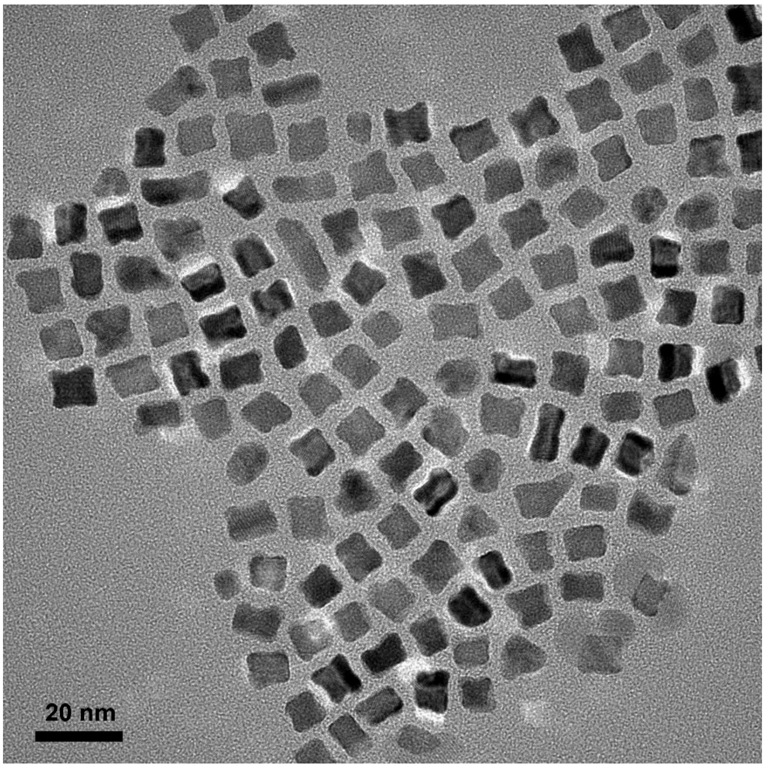

Figure S2. TEM image of Pt nanocubes.

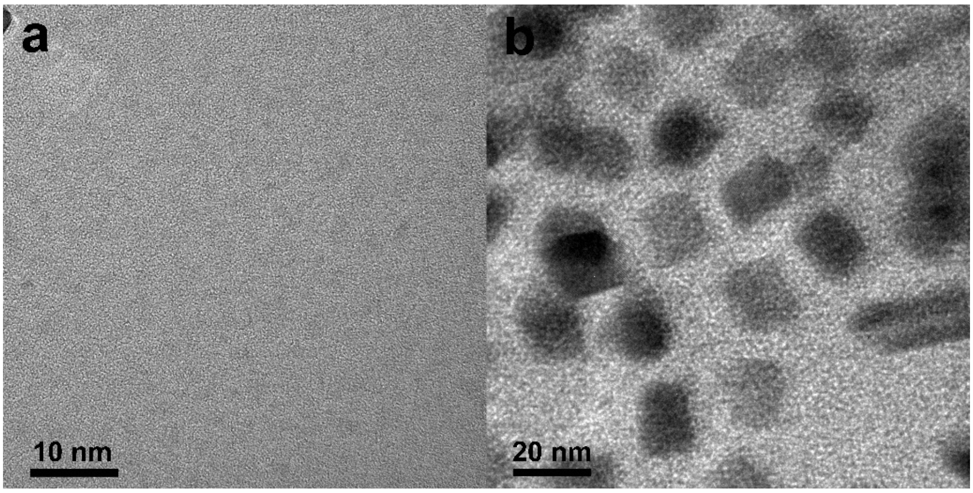

Figure S3. The temporal TEM images of reaction intermediates of Pt-CoO-20 NPs. Before the injection of $Pt(acac)_2$ (a) and 30 min later after injection of $Pt(acac)_2$ (b).

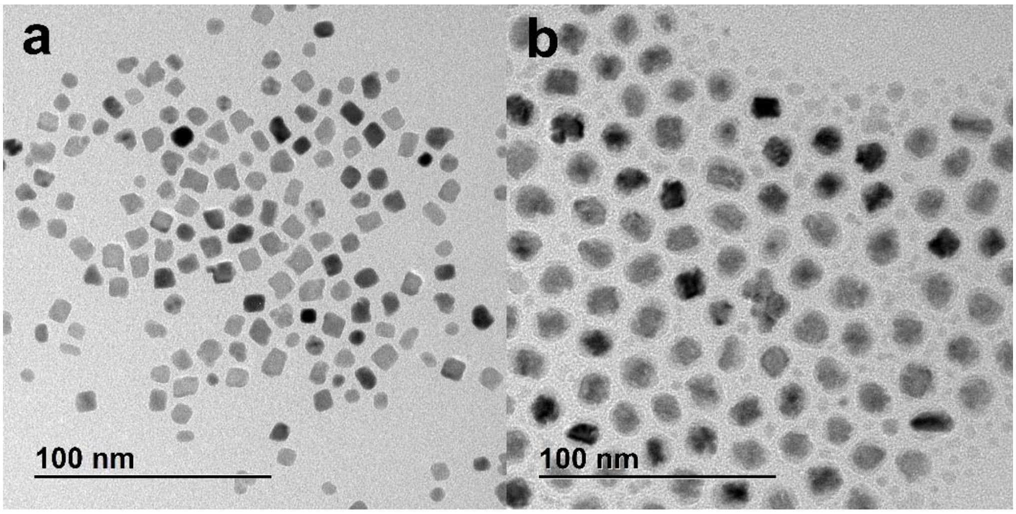

Figure S4. TEM images of Pt-CoO-20 NPs obtained by using absence of OA (a) and adamantaneacetic acid instead of OA (b).

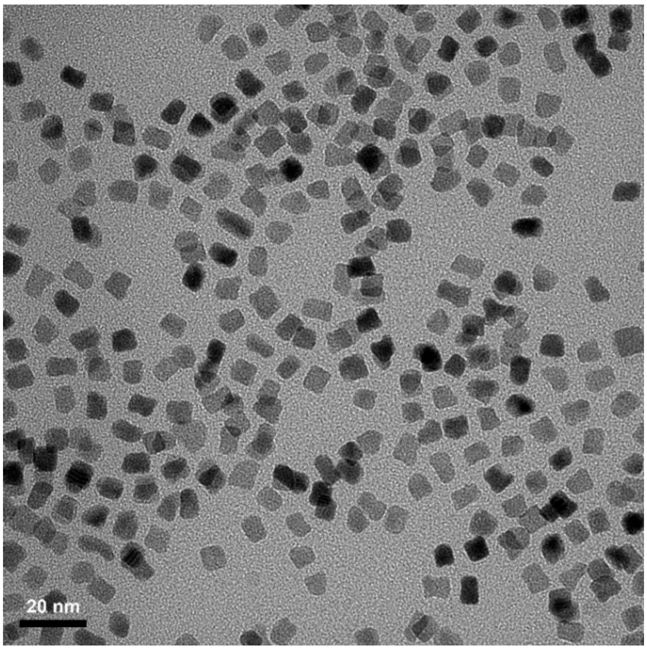

Figure S5. TEM imgaes of Pt-CoO-20 NPs synthesized at 160 °C.

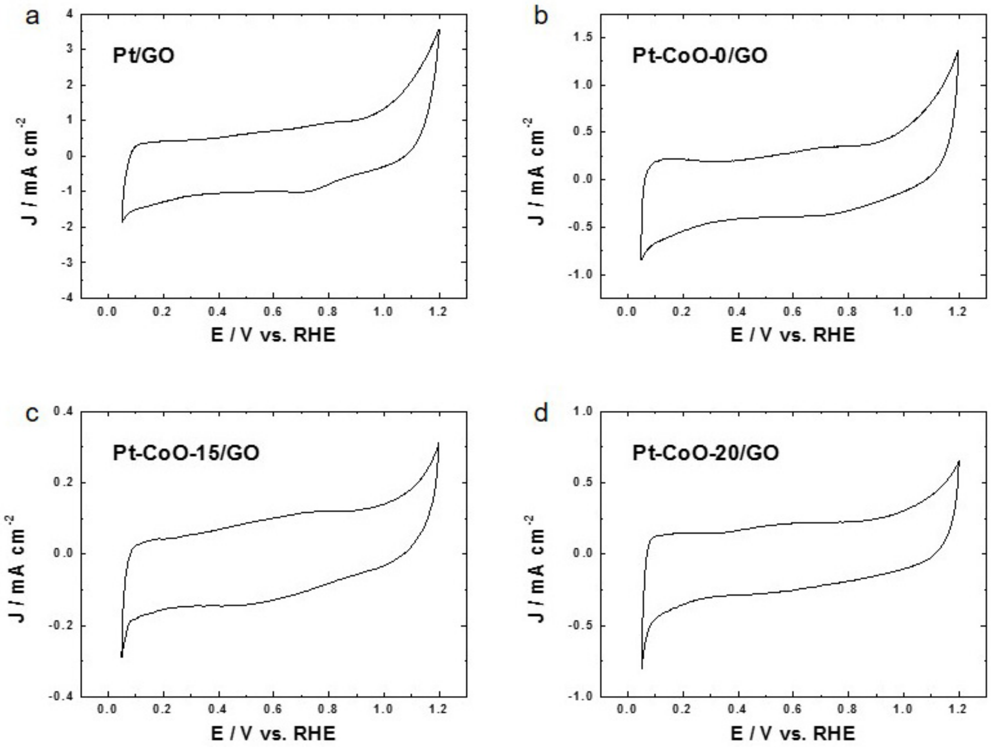

Figure S6. Cyclic voltammograms of Pt/GO, Pt-CoO-0/GO, Pt-CoO-15/GO and Pt-CoO-20/GO.

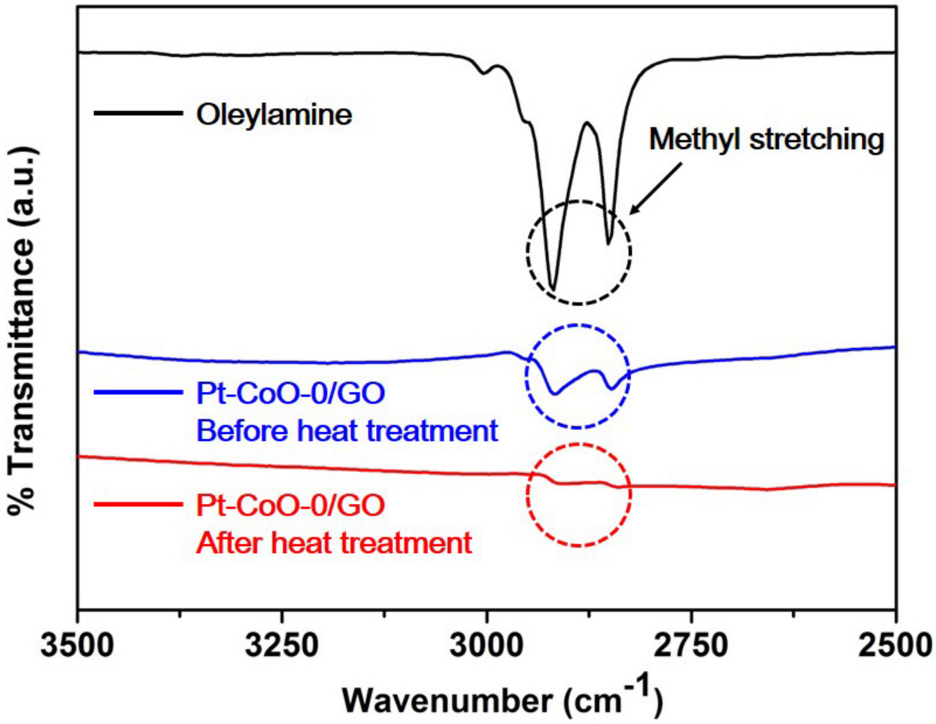

Figure S7. FT-IR spectra of oleylamine (black), Pt-CoO-0/GO before heat treatment (blue) and Pt-CoO-0/GO after heat treatment (red).

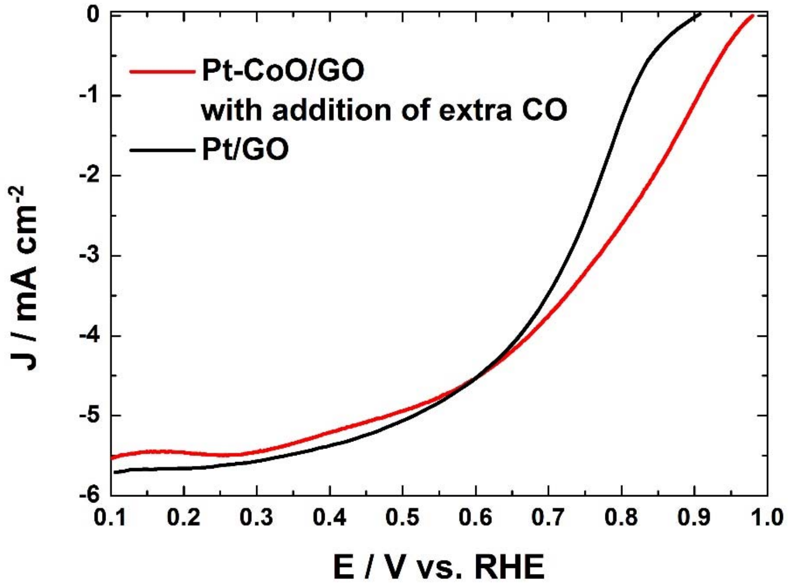

Figure S8. ORR polarization curves at 1600 rpm of Pt-CoO/GO synthesized by addition of extra CO gas (red) and Pt/GO (black).

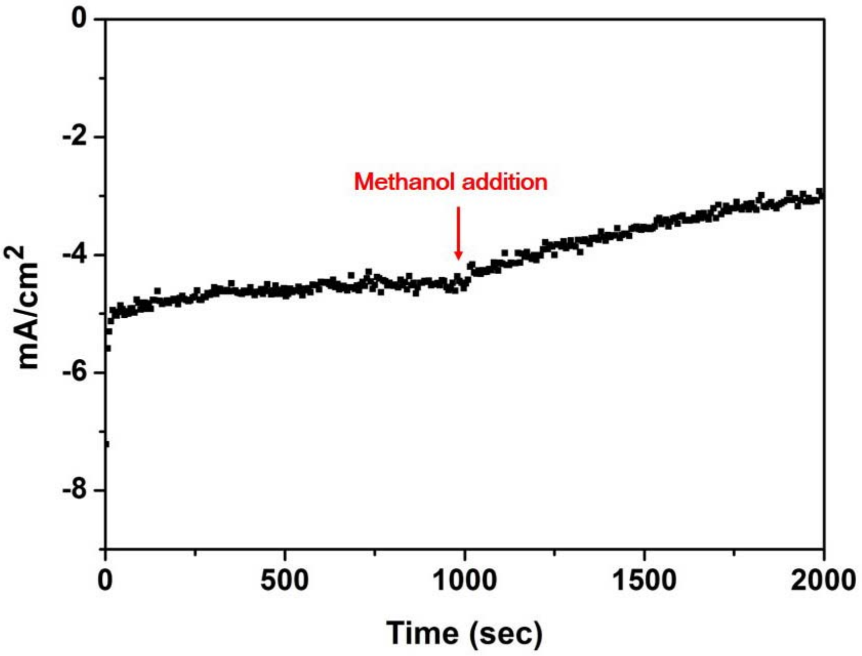

Figure S9. Current–time response for the analysis of methanol poisoning for a fixed potential of 0.05 V (vs. RHE with 1600 rpm rotation rate) at Pt-CoO-0/GO electrodes in 0.1 M $HClO_4$.

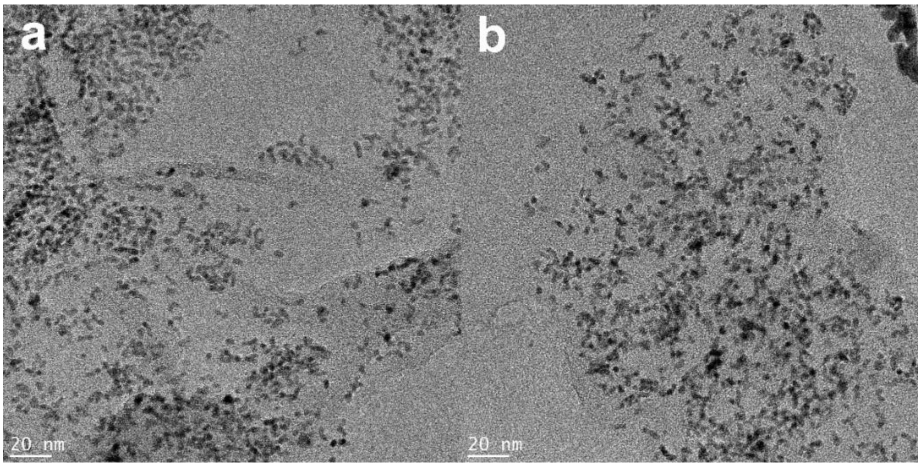

Figure S10. TEM images of before (a) and after (b) long-term stability test.
